# *Rhinolekos capetinga*: a new cascudinho species (Loricariidae, Otothyrinae) from the rio Tocantins basin and comments on its ancestral dispersal route

**DOI:** 10.3897/zookeys.481.8755

**Published:** 2015-02-04

**Authors:** Fábio F. Roxo, Luz E. Ochoa, Gabriel S. C. Silva, Claudio Oliveira

**Affiliations:** 1 Universidade Estadual Paulista, Departamento de Morfologia, Laboratório de Biologia e Genética de Peixes, Rubião Júnior s/n, 18618970 Botucatu, São Paulo State, Brazil

**Keywords:** Biodiversity, Freshwater, Neotropical fish, South America, Taxonomy

## Abstract

The present study deals with the description of a new species of *Rhinolekos*. It can be distinguished from its congeners by having 31 vertebrae, the anterior portion of the compound supraneural-first dorsal-fin proximal radial contacting the neural spine of the 9^th^ vertebra, the absence of transverse dark bands in the pectoral, pelvic and anal-fin rays, 24–28 plates in the dorsal series, the lack of odontodes on the ventral tip of the snout, the absence of accessory teeth, a greater prenasal length, a smaller head length, and by a greater snout length. *Rhinolekos capetinga* is restricted to the headwaters of the rio Tocantins and it is the first species of this genus in the Amazon basin. Additionally, we present a brief discussion of a biogeographic scenario that may explain the dispersal of the new species from the rio Paranaíba to the rio Tocantins basin. We suggest that the ancestral lineage of *Rhinolekos capetinga* reached the rio Tocantins from portions of the rio Paranaíba at the end of the Miocene, about 6.3 Mya (4.1–13.9 Mya 95% HPD), probably as a result of headwater capture processes among adjacent drainages.

## Introduction

Otothyrinae sensu Chiachio et al. (2008) is one of the most diverse and widespread members of Loricariidae, and is composed of about 97 species (Eschmeyer and Fong 2014). Fishes of this subfamily are characterized by morphological specializations such as the presence of metapterygoid channel, the ventral margin of preopercle medially reflected, the rostral plate with posterior notch articulated with mesethmoid, the fourth infraorbital expanded ventrally, and the almost complete fusion of pectoral dermal bony plates forming a strong pectoral armor (Schaefer 1998; Chiachio et al. 2008). In the last fifteen years, new genera and species have been described and assigned (Roxo et al. 2014a) to the subfamily Otothyrinae (e.g. *Gymnotocinclus* Carvalho, Lehmann & Reis, 2008; *Rhinolekos* Martins & Langeani, 2011a), indicating that the great diversity of this group still remains fairly known (Martins and Langeani 2011a).

*Rhinolekos* is the most recently described genus of Otothyrinae and differs from its members, mainly by having the anterior portion of the compound supraneural-first dorsal-fin proximal radial contacting the neural spine of the 9^th^ or 10^th^ vertebrae and by the presence of the lateronasal plate. Currently, *Rhinolekos* includes three valid species: *Rhinolekos britskii* Martins & Langeani, 2011a, *Rhinolekos schaeferi* Martins & Langeani, 2011a and *Rhinolekos garavelloi* Martins & Langeani, 2011a, all of which were described from drainages of the rio Paranaíba (upper rio Paraná basin).

Furthermore, several authors discussed the historical dispersal of the ancient fauna among adjacent drainages of South America Platform (e.g. Ribeiro 2006; Albert and Reis 2011) and specifically species of the families Hypoptopomatinae, Neoplecostominae, and Otothyrinae (e.g. Roxo et al. 2012; Roxo et al. 2014a). The geological process responsible for this distribution pattern is head water captures (also known as stream capture or stream piracy). This is a geomorphological process by which the flow of part of a stream or river drainage basin is diverted into neighboring basin. River capture may facilitate the dispersal of fish species among adjacent drainage and can have profound consequences in isolated local fauna, which watershed boundaries strongly limit their dispersal (Grant et al. 2007; Muneepeerakul et al. 2008; Bertuzzo et al. 2009).

Recently, during collecting expeditions in small tributaries of the rio Tocantins, the major drainage of the Brazilian Shield (Carvalho and Albert 2011), an undescribed species of Otothyrinae, which meets the diagnosis of *Rhinolekos*, was collected and is formally described in the present study. Moreover, we used a time calibrated phylogenetic analysis and parametric biogeographic methods based on available data of Roxo et al. (2014a) to estimate ancestral geographic ranges and hypothesize when the new species reached the rio Tocantins from sections of the rio Paranaíba, probably as a result of head water capture events between these two hydrographic systems in Late Miocene.

## Material and methods

### Morphological analysis

After collection, fish were anesthetized using 1% benzocaine in water, fixed in 10% formaldehyde, and preserved in 70% ethanol for morphological study. Institutional acronyms follow Fricke and Eschmeyer (2014). Vouchers of the morphological study were deposited in the collection of the Laboratório de Biologia e Genética de Peixes (LBP) and Museu de Zoologia da Universidade de São Paulo (MZUSP), Brazil. Measurements and counts were taken on the left side of specimens. Measurements followed Boeseman (1968) with modifications of Armbruster and Page (1996), Schaefer and Provenzano (1993), and Ribeiro et al. (2005) and were taken point to point to the nearest 0.1 mm with digital calipers. Meristic data include numbers of premaxillary and dentary teeth, dorsal, mid-dorsal, median, mid-ventral and ventral plates following Schaefer (1997). Abbreviations used in the text followed Carvalho and Reis (2009). Specimens were cleared and stained (c&s) according to the method of Taylor and Van Dyke (1985). Head plate and osteology nomenclature followed Schaefer (1997). Dorsal-fin ray counts include spinelet as the first unbranched ray. Vertebral counts also include the five vertebrae that comprise the Weberian apparatus. The compound caudal centrum (PU1 + U1) was counted as one element. Zoological nomenclature follows the International Code of Zoological Nomenclature (International Commission on Zoological Nomenclature 1999).

### Molecular analysis

We used *Diplomystes mesembrinus* to root our phylogeny. Additionally, samples of *Corydoras imitator*, *Corydoras oiapoquensis*, *Hoplosternum littorale*, *Callichthys callichthys*, *Astroblepus* sp. 1 and *Astroblepus* sp. 2, *Hemipsilichthys gobio*, *Hemipsilichthys papillatus*, *Delturus parahybae*, *Rineloricaria lanceolata*, *Spatuloricaria* sp. 1, *Hypostomus ancistroides*, *Hypostomus nigromaculatus* and *Hypostomus microstomus* were used as additional outgroups. We included in the analysis 155 specimens representing 115 loricariid species (see Suppl. material 3 – Table S1 to all species names, localities, deposits in museums and GenBank accession numbers).

Vouchers of the molecular study were deposited at the collection of the Laboratório de Biologia e Genética de Peixes (LBP); the Museu de Ciências e Tecnologia, Pontifícia Universidade Católica do Rio Grande do Sul (MCP); the Núcleo de Pesquisas em Limnologia, Ictiologia e Aquicultura (NUP); and the Museum of Natural History of the City of Geneva (MHNG).

## Sequencing

Total DNA was extracted from ethanol preserved muscle samples with the DNeasy Tissue Kit (Qiagen), following manufacturer’s instructions. Partial sequences of the genes 16S rRNA (Kocher et al. 1989), cytochrome *b* (Cytb) (Oliveira et al. 2011), cytochrome c oxidase subunit I (COI) (Ward et al. 2005) and F-reticulon 4 (Chiachio et al. 2008) were amplified using polymerase chain reaction (PCR) with the primers described in Suppl. material 4 – Table S2. Amplifications were performed in a total volume of 12.5 μl with 1.25 μl of 10X buffer (10 mM Tris-HCl+15 mM MgCl2), 0.5 μl dNTPs (200 nM of each), 0.5 μl each 5 mM primer, 0.05 μl Platinum® *Taq* Polymerase (Invitrogen), 1 μl template DNA (12 ng), and 8.7 μl ddH2O. The PCR reactions consisted of 30–40 cycles, 30 s at 95 °C, 15–30 s at 48–58 °C, and 45–90 s at 72 °C. Nested-PCRs were used to amplify the nuclear marker; the first amplification was performed using the primers Freticul4-D and Freticul4-R with a total volume of 12.5 µl for 30–40 cycles (30 s at 95 °C, 30 s at 48 °C, and 135 s at 72 °C); the second amplification was performed using the primers Freticul4 D2 and Freticul4 R2 with a total volume of 12.5 µl for 30–40 cycles (30 s at 95 °C, 30 s at 53–54 °C, and 135 s at 72 °C). All PCR products were first visually identified on a 1% agarose gel and then purified using ExoSap-IT® (USB Corporation) following instructions of the manufacturer. The purified PCR products were sequenced using the “Big DyeTM Terminator v3.1 Cycle Sequencing Ready Reaction Kit” (Applied Biosystems), purified again by ethanol precipitation and loaded on an automatic sequencer 3130-Genetic Analyzer (Applied Biosystems) in the Instituto de Biociências, Universidade Estadual Paulista, Botucatu, São Paulo.

### Phylogenetic analysis

The phylogenetic analysis was performed according to Roxo et al. (2014a) (Suppl. material 1 – Fig. S1). All individual sequences for each species were initially analyzed using the software program BioEdit 5.0.9 (Hall 1999) and consensus sequences were obtained. All sequences for each gene were independently aligned using MUSCLE (Edgar 2004) under default parameters and the alignments inspected by eye for any obvious misalignments. After that, sequences of all genes were concatenated to perform all phylogenetic and biogeography analysis.

Maximum likelihood analyses were performed using RAxML Web-Servers (Stamatakis et al. 2008). RAxML implements a faster algorithm of heuristic searches with bootstrap pseudoreplicates (RBS). Bootstrap (BS) resampling (Felsenstein 1985) was applied to assess support for individual nodes using 1,000 replicates. Random starting trees were used for each independent ML tree search and all other parameters were set on default values.

Bayesian inference (BI) (Huelsenbeck and Ronquist 2001) was performed evaluating alternative tree topologies through the estimation of posterior probabilities (P) using MrBayes v.3.0 (Ronquist and Huelsenbeck 2003). The ML tree obtained from ML analysis was used as a starting three for the Markov chain Monte Carlo searches. Eight chains were run simultaneously for 100,000,000 generations and every 1000^th^ generation a tree was sampled. The above analysis was performed twice. The distribution of log-likelihood scores was examined to determine stationary phase for each search and to decide if extra runs were required to achieve convergence, using the program Tracer 1.5 (Rambaut and Drummond 2007a). All sampled topologies beneath the asymptote (25,000,000 generations) were discarded as part of a burn-in procedure, and the remaining trees were used to construct a 50% majority-rule consensus tree in TreeAnnotator v1.7.5 (Rambaut and Drummond 2007b).

### Time calibrated phylogeny and hypothesis on the ancestor

The time calibrated phylogeny was performed according to Roxo et al. (2014a) (Suppl. material 2 – Fig. S2). The uncorrelated relaxed molecular clock was calibrated using BEAST (Bayesian Evolutionary Analysis Sampling Trees) v1.6.2 (Drummond and Rambaut 2007). Two fossil calibration points were used to constrain divergence times for all clades of the phylogenetic tree. The first calibration point was implemented as a normally distributed prior, with an offset of 125 million years ago (Ma), and a standard deviation of 15 million years. These date-estimate parameters were selected to match current knowledge of the timing of siluriform origins. Information from the stratigraphic record and geographic distributions of living taxa indicate an origin for Siluriformes during the Lower Cretaceous (145–100 Ma; Lundberg 1993; Sullivan et al. 2006; Lundberg et al. 2007). We used a birth–death model for speciation likelihood and a starting tree obtained from ML. The analysis was run for 100 million generations and sampled every 1000^th^ generation. Stationarity and sufficient mixing of parameters (ESS>200) was checked using Tracer v1.5 (Rambaut and Drummond 2007a). A consensus tree was built using TreeAnnotator v1.6.2 (Rambaut and Drummond 2007b).

Data on the geographic distributions of species were taken from the original species descriptions and information available at the Catalog of Eschmeyer (2014). We assigned taxa to geographic areas using the ecoregion classifications of Vari and Malabarba (1998) and Chiachio et al. (2008), within the following five biogeographic regions: A, Atlantic Coastal Drainages of Southeastern Brazil; B, Upper Paraná Basin; C, Paraguay, Lower Paraná and Uruguay basins; D, Amazon and Orinoco basins; E, São Francisco basin and Coastal Drainages of Northeastern of Brazil (see Roxo et al. 2014a for more details about biogeographic area classifications). The new species *Rhinolekos capetinga* is assigned to the D area (Amazon and Orinoco basins) in the present paper.

The maximum-likelihood analysis of biogeographic history was performed in Lagrange v2.0 (Ree et al. 2005; Ree and Smith 2008) using all available data and parameters of Roxo et al. (2014a). Four DEC models were tested to estimate distribution ranges inherited by the descending lineages at each node of the tree. The differences between the models are in the rate of dispersal among adjacent and no adjacent areas (see Suppl. material 5 – Table S3 for the likelihood values and dispersal rate among adjacent and no adjacent areas for each model). The model that obtained the highest ML values was model 3 (M3) that constrained the dispersal rates between adjacent areas at 0.5 and areas separated by one or more intercalated areas at 0.0001.

## Results

### Rhinolekos capetinga sp. n.

Taxon classificationAnimaliaSiluriformesLoricariidae

http://zoobank.org/53CB690E-E969-4C06-8C1E-4991C103F19F

**Figure 1.**
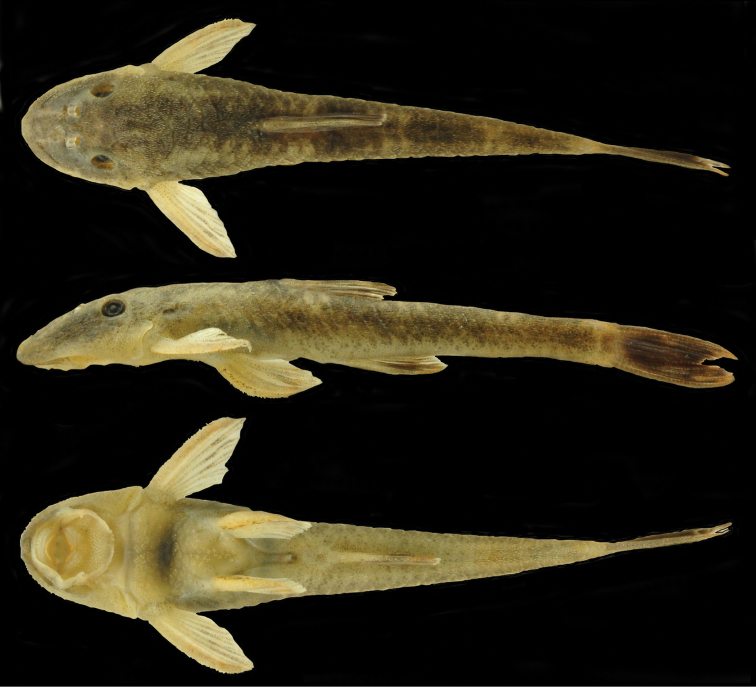
*Rhinolekos capetinga* MZUSP 116102, holotype, male, 37.5 mm SL, Goiás State, rio Tocantins basin, Brazil.

Figs 1 , 3 ; Table 1

Rhinolekos sp. 1 – Roxo et al. 2014a: 9(8) e105564 (phylogenetic relationships).

#### Holotype.

MZUSP 116102, (male, 37.5 mm SL), Brazil, Goiás State, municipality of Água Fria de Goiás, córrego da Branca, drainage of the rio Tocantizinho, rio Tocantins basin, 14°53'47.2"S, 47°34'58.4"W, 30 June 2014, FF Roxo, GSC Silva, LE Ochoa.

#### Paratypes.

Brazil, Goiás State, rio Tocantins basin (56 specimens). LBP 17089 (1 male, 39.1 mm SL), municipality of Agua Fria de Goiás, córrego da Branca, drainage of the rio Tocantizinho, 14°57'01.6"S, 47°35'57.0"W, 21 November 2012, R Devidé, BF Melo, JMH Martinez, GSC Silva; LBP 18996, (1 female, 24.1 mm SL), municipality of São João D’Aliança, córrego Roncador, drainage of the rio Tocantizinho, 14°43'51.3"S, 47°32'34.0"W, 30 June 2014, FF Roxo, GSC Silva, LE Ochoa; LBP 19001 (15 females, 26.8–36.2 mm SL, 20 males, 39.5–30.2 mm SL, 3 c&s, 37.2–32.6 mm SL, 9 unsexed juveniles not measured), collected with holotype. LBP 19466 (2 females, 36.5–37.1 mm SL) municipality of Água Fria de Goiás, córrego da Branca, drainage of the rio Tocantizinho, 14°53'47.2"S, 47°34'58.4"W, 09 November 2014, FF Roxo, LH Roxo, GSC Silva, LE Ochoa; MZUSP 113920 (2 females, 29.3–37.3 mm SL, 3 males, 30.4–39.0 mm SL), municipality of Água Fria de Goiás, córrego da Branca, drainage of the rio Tocantizinho, 14°53'47.2"S, 47°34'58.4"W, 27 November 2012, OT Oyakawa, AM Zanata, P Camelier, M Melo.

#### Diagnosis.

**Figure 2.**
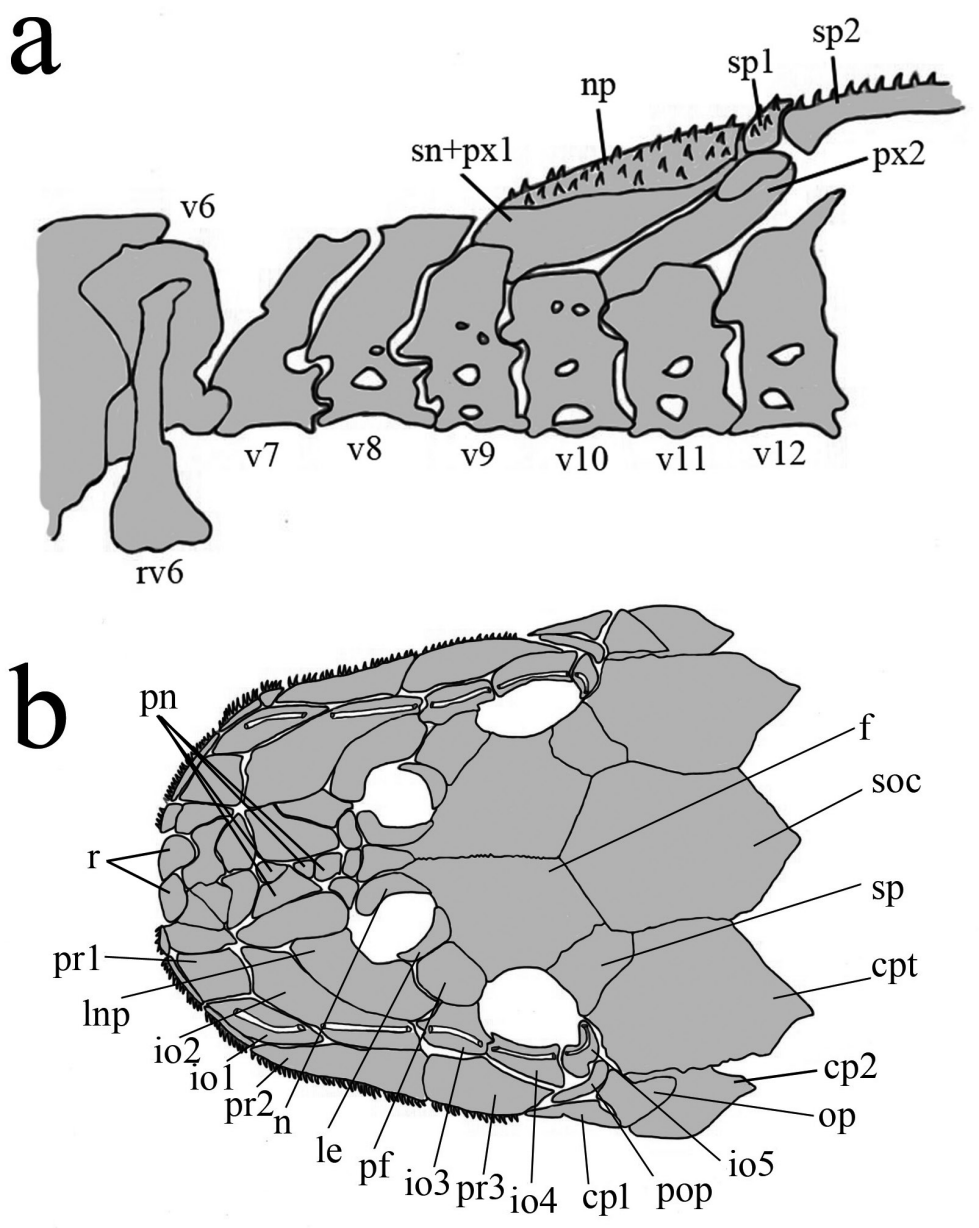
*Rhinolekos capetinga*, LBP 19001, paratype, 34.5 mm SL. **a** Anterior portion of axial skeleton and dorsal-fin supports (left side, lateral view). Vertebrae counts included five vertebrae of the Weberian apparatus. **np** nucal plate; **rv6** rib of sixth vertebrae; **px2** compound proximal and medial radial 2; **sn+px1** compound supraneural first dorsal-fin proximal radial; **sp1** first dorsal-fin spinelet; **sp2** second dorsal-fin spine; **v6−12** vertebrae 6−12 **b** Skull of *Rhinolekos capetinga*; **f** frontal; **soc** supraoccipital; **cpt** parieto-supraoccipital; **op** opercle; **io1−5** infraorbitals; **pop** preopercle; **cp 1−2** cheek plates; **pr 1−3** postrostral plates; **pf** prefrontal plates; **le** lateral ethmoid; **n** nasal; **lpn** lateronasal plate; **r** rostral plate; **pn** prenasal; **sp** sphenotic.

*Rhinolekos capetinga* differs from *Rhinolekos garavelloi* and *Rhinolekos schaeferi* in that it has a lower number of vertebrae, 31 (vs. 32) and the anterior portion of the compound supraneural-first dorsal-fin proximal radial contacts the neural spine at the 9^th^ vertebra (vs. 10^th^, Fig. 2a). The new species can be distinguished from *Rhinolekos britskii* by the absence of transverse dark bands in the pectoral, pelvic and anal-fin rays (vs. present), lower number of plates in the dorsal series 24–28 (vs. 30–35), lack of odontodes on the ventral tip of the snout (vs. tip of snout completely covered by odontodes), and by having a greater prenasal length, 41–60% of HL (vs. 32–40% of HL). Moreover, the new species differs from *Rhinolekos schaeferi* by the absence of accessory teeth (vs. present) and from all congeners by the smaller head length, 20–27% of SL (vs. 28–32% of SL in *Rhinolekos britskii*; 29–35% of SL in *Rhinolekos garavelloi*; 29–32% of SL in *Rhinolekos schaeferi*), and by the greater snout length, 61–85% of HL (vs. 52–57% of SL in *Rhinolekos britskii*; 49–60% of SL in *Rhinolekos garavelloi*; 53–59% of SL in *Rhinolekos schaeferi*). It differs from *Rhinolekos britskii* and *Rhinolekos garavelloi* by the smaller caudal-peduncle depth, 6–9% of SL (vs. 9–11% of SL in *Rhinolekos britskii* and 10–13% of SL in *Rhinolekos garavelloi*); it differs from *Rhinolekos garavelloi* by the smaller thoracic length 10–15% of SL (vs. 18–21% of SL), and by the smaller folded dorsal-fin length, 14–21% of SL (vs. 22–26% of SL).

#### Description.

Morphometric and meristic data presented in Table 1. Maximum body length 39.1 mm SL; dorsal profile of head in lateral view convex to straight from upper part of rostrum to anterior margin of eyes, slightly curved from eyes to posterior margin of parieto supraoccipital, almost straight to dorsal-fin origin; dorsal profile of trunk almost straight, descending from base of dorsal-fin origin to caudal peduncle; ventral profile slightly concave from snout tip to pelvic-fin origin, slightly convex to caudal peduncle; greatest body depth at dorsal-fin origin; greatest body width at cleithral region, gradually decreasing towards snout and caudal-fin. Cross-section of caudal peduncle almost ellipsoid; rounded laterally and almost flat dorsally and ventrally.

**Table 1.** Morphometrics and meristic data for *Rhinolekos capetinga*. SD, standard deviation.

	*Rhinolekos capetinga*, holotype and paratypes (n=30)			
	Holotype	Range	Mean	SD
**SL**	**37.5**	**22.9–39.1**	**34.3**	**3.6**
**Percents of SL**				
Predorsal length	42.2	38.9−49.9	44.7	2.0
Preanal length	53.4	48.2−60.3	54.0	2.7
Prepectoral length	26.2	19.1−26.2	23.2	1.4
Prepelvic length	33.3	31.5−39.5	35.2	2.0
Postanal length	34.4	28.0−38.7	34.8	2.5
Thoracic length	13.1	9.6−15.2	12.9	1.5
Abdominal Length	19.7	11.8−23.5	19.2	2.2
Caudal peduncle depth	6.6	5.8−8.6	6.9	0.6
Head length	21.6	19.6−26.6	22.4	1.5
Head width	22.1	17.6−26.6	21.8	1.8
Head depth	11.9	10.8−15.7	12.8	1.0
Base of dorsal-fin length	10.9	9.3−13.0	10.5	1.0
Folded dorsal-fin length	20.7	13.9−21.3	19.1	1.4
Pectoral-fin unbranched ray length	20.1	13.6−22.9	19.9	1.9
Pelvic-fin unbranched ray length	15.3	13.3−17.5	15.6	1.2
Snout-opercle length	21.8	18.8−26.3	21.9	1.5
**Percents of HL**				
Snout length	60.9	60.7−85.2	72.4	5.8
Orbital diameter	19.6	12.2−23.2	17.1	2.3
Interorbital length	45.4	40.4−55.8	46.6	3.8
Prenasal length	48.8	41.3−60.2	51.4	4.4
Suborbital depth	26.3	19.0−39.7	25.6	4.5
**Meristics**	**Holotype**	**Range**	**Mode**	**SD**
Left premaxillary teeth	26	15−34	22	–
Left dentary teeth	24	14−31	26	–
Dorsal plates	27	24−28	27	–
Mid-dorsal plates	17	16−20	18	–
Median plates	25	23−27	25	–
Mid-ventral plates	25	20−24	22	–
Ventral plates	18	15−18	17	–

Head rounded in dorsal view. Snout slightly pointed, its tip rounded, elongated (61–85% of HL) and depressed in front of each nostril on dorsal surface. Anterior margin of snout covered with odontodes, except ventral tip of snout; odontodes of margin of snout similar in size to remaining ones found on head. Odontodes on head and trunk well defined and not forming longitudinal rows; eye small (12–23% of HL), dorsolaterally positioned; iris operculum not present; lips roundish and papillose; papillae uniformly distributed on base of dentary and premaxillary and slightly decreasing distally. Lower lip larger than upper lip; its border fringed; maxillary barbel present; Teeth slender and bicuspid; mesial cusp larger than lateral cusp; premaxillary teeth 15–34. Dentary teeth 14–30.

Dorsal fin ii,6-7; dorsal-fin spinelet short, roughly triangular shaped, locking mechanism non-functional; dorsal-fin origin slightly posterior of vertical through pelvic-fin origin. Anterior portion of compound supraneural-first dorsal-fin proximal radial contacting neural spine of 9^th^ vertebrae (Fig. 2a). Tip of adpressed dorsal-fin rays slightly surpassing end of anal-fin base. Pectoral fin i,5-6; tip of longest pectoral-fin ray almost reaching to middle of adpressed pelvic-fin, when depressed. Pectoral axillary slit not present even in juveniles. Pectoral spine supporting odontodes anteroventrally; pelvic fin i,5; its tip not exceeding anal-fin origin when depressed in both sexes. Pelvic-fin unbranched ray with dermal flap along its dorsal surface in males; anal fin i,5; its tip reaching 7^th^ and 8^th^ plate from its origin; Caudal fin i,14,i; distal margin forked; Adipose-fin absent. Total vertebrae 31 (3 c&s).

Body covered with bony plates except on ventral part of head, around pectoral and pelvic-fin origin and on dorsal-fin base. Cleithrum and coracoid totally exposed; Arrector fossae partially enclosed by ventral lamina of coracoids. Abdomen entirely covered by plates in adults (about 25.0 mm SL); lateral plate series with elongate and large plates, formed by two lateral plate series, similar in size; median plates formed by four to five irregular plate series reaching anal shield. Lateral side of body entirely covered by plates; mid-dorsal and mid-ventral plates well developed, reaching typical adipose-fin region.

Parts of head osteology presented in Fig. 2b. Tip of snout formed by two square rostral plates. Nasal almost rectangular forming anterior medial nostril margin in contact posteriorly with frontals, and anterior and lateral margins contacting pre-nasals. Lateral surface of head formed by three posterior rostrum plates, second one large and triangular shaped. Complete infraorbital plate series, present mesial to posterior rostrum series, composed of five plates; fourth infraorbital expanded ventrally, all associated with latero-sensory canal system; first and second infraorbitals largest and fifth smallest. Large lateronasal plate mesial to second infraorbital, forming anterior distal nostril margin in contact anteriorly with prenasals and posteriorly with prefrontal. Preopercle present just ventral to fifth infraorbital; an elongated bone covered by latero-sensory canal. Subocular cheek plates present ventral to preopercle plate. Top of head composed of compound pterotic-supracleithrum, supraoccipital, prefrontal, frontal, and sphenotic; parieto-supraoccipital bearing fenestrae irregularly distributed and of different sizes and shapes.

#### Color in life.

**Figure 3.**
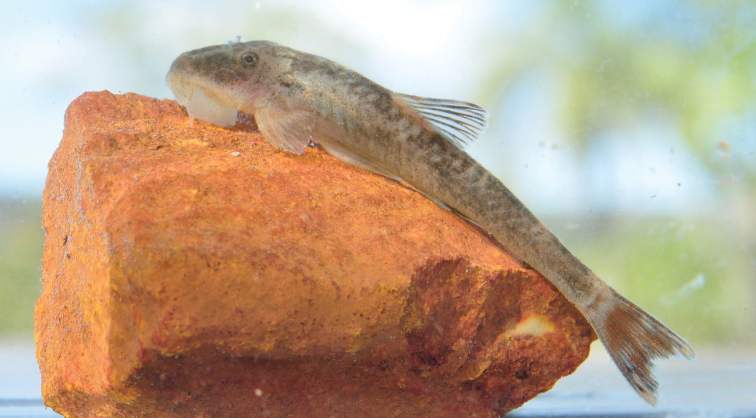
*Rhinolekos capetinga*, live specimen, MZUSP 116102, holotype, male, 37.5 mm SL, rio Tocantins basin, Goiás State, Brazil. Photo: FF Roxo.

Pale yellowish ground color. Dorsal surface of head dark brown, except for pale yellowish areas on snout tip. Four dark-brown saddles crossing dorsum, reaching longitudinal dark strip on side of trunk: first at dorsal-fin origin, second below dorsal-fin base, third typically at adipose-fin region, and fourth at end of caudal peduncle. Caudal-fin black, with small hyaline circular area on each lobe, tip of lobes hyaline; some specimens with caudal-fin lobe entirely dark (Fig. 3).

#### Color in alcohol.

Similar pattern described for living specimens, but with ground color dark brown (Fig. 1).

#### Sexual dimorphism.

Adult males are distinguished by having a papilla at the urogenital opening (vs. papilla absent in females), and by an unbranched pectoral- and pelvic-fin ray supporting a dermal flap on their proximal dorsal surface in males.

#### Etymology.

The specific name *capetinga* from the Tupi-guarani dialect is in reference to the old and unused name of São João D´Aliança municipality. The name «capetinga» means white, or clear water. A noun in apposition.

#### Distribution.

**Figure 4.**
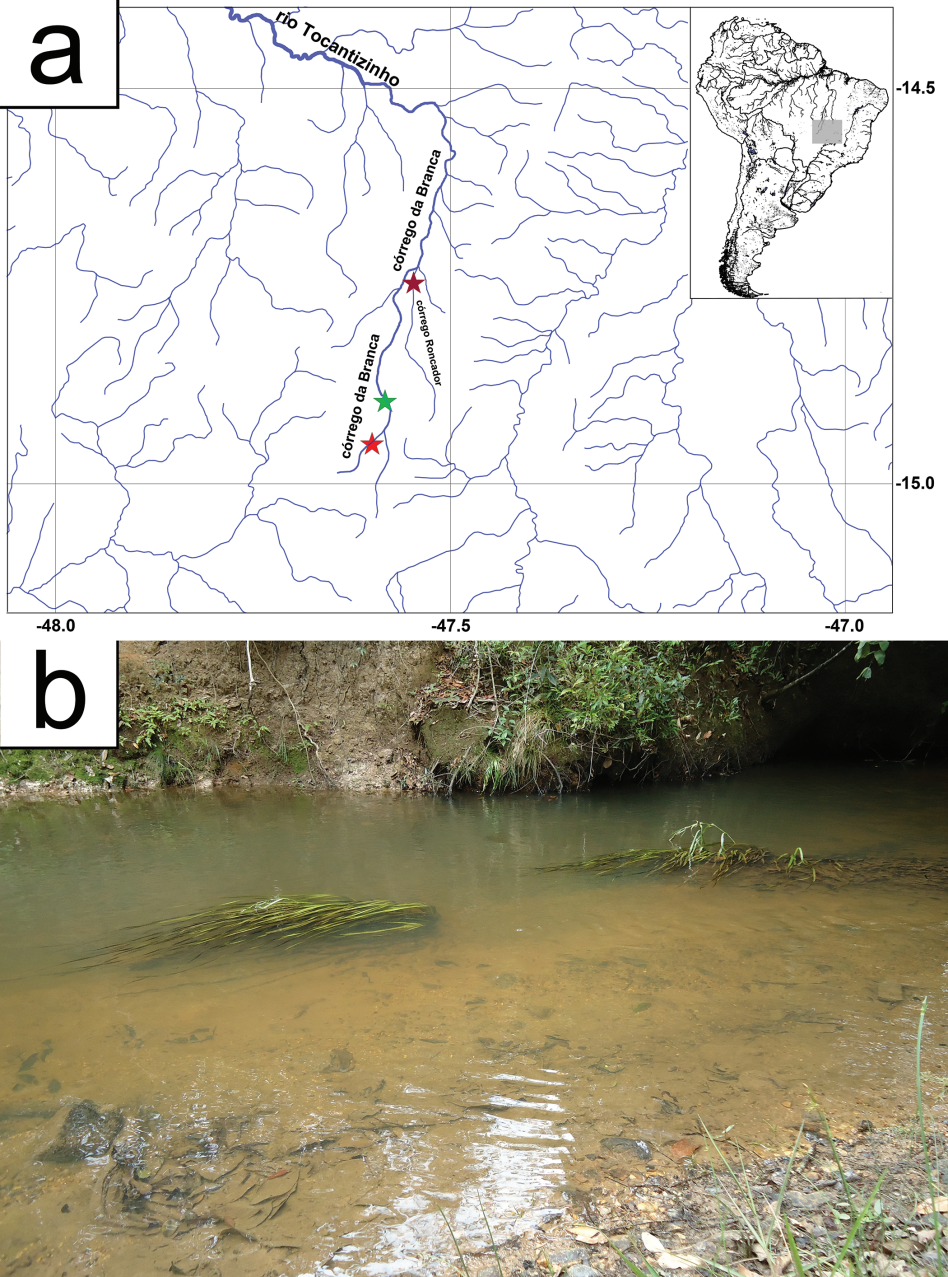
**a** Map showing the distribution of *Rhinolekos capetinga*. Type locality at córrego da Branca, green star – 14°53'47.2"S, 47°34'58.4"W. Paratype localities at córrego da Branca, red star – 14°57'01.6"S, 47°35'57.0"W, and at córrego Roncador, pink star – 14°43'51.3"S, 47°32'34.0"W **b** Habitat and submerged vegetation where the specimens were found in type locality of córrego da Branca, 14°53'47.2"S, 47°34'58.4"W. Photo: LH Roxo.

*Rhinolekos capetinga* is known from two localities at the córrego da Branca and one locality at the córrego Roncador, all drainages of the rio Tocantizinho, rio Tocantins basin (Fig. 4a).

#### Habitat.

*Rhinolekos capetinga* was collected on flat areas of the córrego da Branca and córrego Roncador, rio Tocantins basin, in places of shallow clear waters, about 1 m depth and median to fast current flow. The fishes captured were associated with the vegetation that covers the bottom and the border of the headwaters (Fig. 4b).

##### Phylogenetic and time calibrated tree

Partial sequences of the three mitochondrial genes (16S rRNA, COI, Cytb) and one nuclear gene (F-reticulon 4) were obtained from GenBank (Suppl. material 3 – Table S1) (same data available in Roxo et al. 2014a). The combined sequence data resulted in a matrix of 4,500 base pairs. This matrix was used to perform all phylogenetic and biogeographic analyses. Bayesian and ML phylogenetic analyses resulted in very similar topologies (Suppl. material 1 – Fig. S1). Our results illustrate the same phylogenetic relationship of Roxo et al. (2014) that the clades Hypoptopomatinae, Neoplecostominae and Otothyrinae are monophyletic with strong statistical support (BS = 96, P = 0.99 for Hypoptopomatinae; BS = 99, P = 1.00 for Neoplecostominae; BS = 96, P = 0.99 with BI for Otothyrinae). The new species *Rhinolekos capetinga* formed sister group to the species *Rhinolekos garavelloi*, and both species formed sister group to the species *Rhinolekos britskii*.

**Figure 5.**
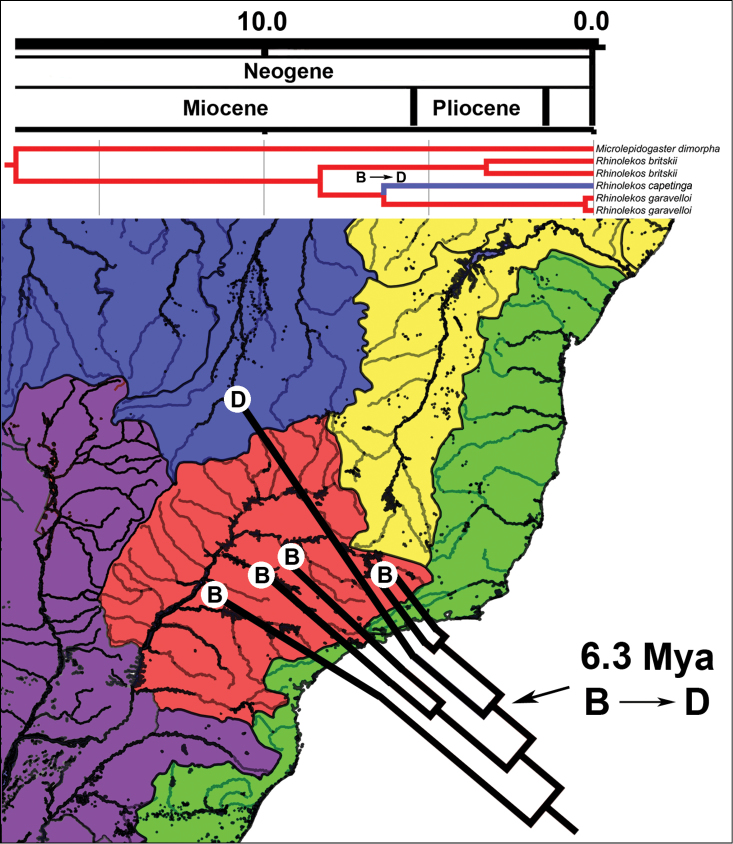
Biogeographic distribution and time-calibrated phylogenetic tree of *Microlepidogaster* and *Rhinolekos* species, based on three mitochondrial (16SrRNA, COI, Cytb) and one nuclear marker (F-reticulon 4), modified from figure 7 of Roxo et al. (2014a). The map colorations indicate distinct biogeographic regions according to classification available in Roxo et al. (2014a): Green – Coastal drainages (**A**); Red – upper rio Paraná basin (**B**); Purple – Paraguay, Lower Paraná and Uruguay basins (**C**); Blue – Amazon basin (**D**); Yellow – São Francisco basin (**E**).

Our time calibrated phylogeny and the ancestral area reconstruction (Suppl. material 2 – Fig. S2; Fig. 5) suggested that the genus *Rhinolekos* originated in the upper rio Paraná basin about 17.5 Mya (9.6–27.9 Mya 95% HPD) and the new species *Rhinolekos capetinga* reached the area D (Amazon and Orinoco basins) from drainages of the rio Paranaíba about 6.3 Mya (4.1–13.9 Mya 95% HPD) at the end of Miocene.

## Discussion

The new species *Rhinolekos capetinga* is a typical species of the genus, given that it presents the main characters used by Martins and Langeani (2011a): the lateronasal plate just above second infraorbital, forming anterior distal nostril margin in contact anteriorly with prenasals, and the anterior portion of the compound supraneural-first dorsal-fin proximal radial contacting the neural spine of the 9^th^ vertebrae, a character that, according to Schaefer (1998), is a homoplastic condition evolved independently many times among the Otothyrinae.

Martins and Langeani (2011a) suggested that the presence of the lateronasal plate is a character state that is present not only in *Rhinolekos* but also shared among species of *Acestridium* Haseman, 1911 and *Gymnotocinclus*. The works of Cramer et al. (2011) and Roxo et al. (2014a) suggested that species of these two genera are not closely related to *Rhinolekos*. *Gymnotocinclus* was found to be the sister group to the genus *Corumbataia* Britski, 1997, and *Acestridium* was included in a polytomy with other Otothyrinae species (Cramer et al. 2011). In contrast, *Acestridium* was found to be the sister group to *Hypoptopoma* Günther, 1868 by Roxo et al. (2014a). Considering these hypotheses, the lateronasal plate, the main character used to distinguish *Rhinolekos* from *Microlepidogaster* Eigenmann & Eigenmann, 1889, as proposed by Martins et al. (2011a), is a homoplasy.

The anterior portion of the compound supraneural-first dorsal fin proximal radial contacting the neural spine of the 9^th^ vertebrae (Fig. 2a) is present in the new species *Rhinolekos capetinga* and that character state is shared with *Rhinolekos britskii*, the most similar species externally. However, in a phylogenetic and biogeographic study of Hypoptopomatinae, Neoplecostominae, and Otothyrinae, Roxo et al. (2014a) suggested that *Rhinolekos capetinga* (identified in that study as *Rhinolekos* sp. 1) form a sister group with *Rhinolekos garavelloi*, and both species form a sister group with *Rhinolekos britskii*. This result suggests that the compound supraneural-first dorsal fin proximal radial contacting the neural spine of the 9^th^ vertebrae is a homoplasy, since that in *Rhinolekos garavelloi* and *Rhinolekos schaeferi* it contacting the neural spine of the 10^th^ vertebrae. Furthermore, in all other Hypoptopomatinae and Otothyrinae species the compound supraneural-first dorsal fin-proximal radial contacting the neural spine of the 7^th^ vertebrae and in *Epactionotus* contacting the neural spine of the 8^th^ vertebrae (Martins and Langeani 2011a).

Martins et al. (2014a) reported that the pectoral-fin axillary slit is present in many species of Otothyrinae. Within *Rhinolekos*, the slit was reported to be absent in adults, but present in juveniles (Martins and Langeani 2011a). However, it was not observed in the new species, even in very young specimens. The complete absence of the pectoral-fin axillary slit is a condition shared with *Otothyris travassosi* Garavello, Britski & Schaefer, 1998, *Otothyris rostrata* Garavello, Britski & Schaefer, 1998, *Otothyris lophophanes* (Eigenmann & Eigenmann, 1889), *Otothyris juquiae* Garavello, Britski & Schaefer, 1998, and *Schizolecis guntheri* (Miranda Ribeiro, 1918) (Martins et al. 2014a), and, according to Reis and Schaefer (1998), its presence is a derived condition in Otothyrinae and its absence is a secondarily derived condition.

### Biogeography and geodispersal route

*Rhinolekos capetinga* is the first species of *Rhinolekos* described in the rio Tocantins basin. Results of Roxo et al. (2014a) suggested that this genus originated in the upper rio Paraná basin at 17.5 Mya (9.6–27.9 Mya 95% HPD) (also see Suppl. material 2 – Fig. S2 of the present study). However, in last paper the species *Rhinolekos capetinga* (*Rhinolekos* sp. 1 in Roxo et al. 2014a) was erroneously assigned to the B area (upper Paraná River basin). In our study we corrected this misunderstanding and assigned the species *Rhinolekos capetinga* to the D area (Amazon and Orinoco basins) during the ancestral area estimation performed in Lagrange and found that dispersal events in the end of the Miocene, about 6.3 Mya (4.1–13.9 Mya 95% HPD), let the ancestor of *Rhinolekos capetinga* reach the rio Tocantins basin (Suppl. material 2 – Fig. S2; Fig. 5) from drainages of rio Paranaíba basin.

Several authors (e.g. Eigenmann and Eigenmann 1891; Jordan 1896; Pearson 1937; Carvalho and Albert 2011; Ribeiro et al. 2013) suggested that headwater captures could explain the movement of fish lineages among the rio Paraguay, the Amazon River tributaries (e.g. Madeira, Tocantins and Xingu) and the drainages of the Brazilian Shield (e.g. rio Paranaíba). By definition, headwater captures change the spatial location of a watershed acting simultaneously as a vicariant process and occasioning biological dispersal. This geological process has been widely reported as responsible for fish movement among adjacent drainages (Ribeiro 2006; Albert et al. 2011; Carvalho and Albert 2011; Roxo et al. 2012; Roxo et al. 2014b), and thus for fish movements of the subfamily Otothyrinae (Roxo et al. 2014a).

Lima and Ribeiro (2011) substantiated that the ichthyofauna of the rio Tocantins shares species with the trans-boundary river basins, namely the Paraguay, Paraná, São Francisco, and Xingu drainages. Additionally, Montoya-Burgos (2003) also associated cladogenetic events within species of *Hypostomus* Lacepède, 1803 with divisions among the rio Amazon tributaries and the Paraguay-Paraná system. Therefore, considering the previous hypothesis, we believe that headwater captures may have influenced the movement of ancestral species of *Rhinolekos capetinga* from the drainages of the rio Paranaíba to the rio Tocantins at the end of the Miocene (Fig. 5).

### Comparative material

*Microlepidogaster arachas* Martins, Calegari & Langeani, 2013: LBP 10882, 3, 22.3−36.3 mm SL, rio Araguari, rio Paranaíba basin; LBP 11724, 9, 38.0−41.2 mm SL, 1 c&s, 39.1 mm SL, córrego sem nome, rio Paranaíba basin; *Microlepidogaster discus* Martins, Rosa & Langeani, 2014b: MZUSP 115384, 2, 38.8−40.4 mm SL, rio Itacambiruçu, rio Jequitinhonha basin; *Microlepidogaster dimorpha* Martins & Langeani, 2011b: LBP 10683, 2, 28.8−35.6 mm SL, rio Uberaba, upper rio Paraná basin; *Microlepidogaster longicolla* Calegari & Reis, 2010: LBP 17077, 4, 39.7−46.4 mm SL, rio Pipiripari, upper rio Paranaíba basin; LBP 17060, 39.1−40.2 mm SL, córrego Maria Velha, upper rio Paranaíba basin; *Microlepidogaster perforatus* Eigenmann & Eigenmann, 1889: LBP 19498, 1, 28.9 mm SL, rio Carandaí, rio São Francisco basin; *Rhinolekos britskii* Martins & Langeani, 2011a: LBP 7245, 3, 28.9−30.5 mm SL, rio Arapuca, rio Paranaíba basin; LBP 7253, 15, 21.7−35.2 mm SL, córrego sem nome, rio Paranaíba basin; MZUSP 103698, 6 paratypes, 27.1−36.1 mm SL, córrego sem nome, rio Paranaíba basin; *Rhinolekos garavelloi* Martins & Langeani, 2011a: LBP 7246, 24, 24.1−34.8 mm SL, córrego Fazenda Lageado, rio Paranaíba basin; MZUSP 103697, 5 paratypes, 21.4−31.9 mm SL, córrego da Fazenda Lageado, rio Paranaíba basin; *Rhinolekos schaeferi* Martins & Langeani, 2011a: LBP 19460, 1, 28.5 mm SL, córrego Fazenda Garaíbas, rio Paranaíba basin; LBP 19461, 1, 36.6 mm SL, córrego Fazenda Garaíbas, rio Paranaíba basin; *Rhinolekos* sp.: LBP 7247, 26, 24.1−33.1 mm SL, córrego Fazenda Balsamo, rio Paranaíba basin.

## Supplementary Material

XML Treatment for Rhinolekos capetinga

## Supplementary materials

Supplementary material 1 Fig. S1 Data type: Adobe PDF file This dataset is made available under the Open Database License (http://opendatacommons.org/licenses/odbl/1.0/). The Open Database License (ODbL) is a license agreement intended to allow users to freely share, modify, and use this Dataset while maintaining this same freedom for others, provided that the original source and author(s) are credited.Fábio F. Roxo, Luz E. Ochoa, Gabriel S. C. Silva, Claudio Oliveira

Supplementary material 2 Fig. S2 Data type: Adobe PDF file This dataset is made available under the Open Database License (http://opendatacommons.org/licenses/odbl/1.0/). The Open Database License (ODbL) is a license agreement intended to allow users to freely share, modify, and use this Dataset while maintaining this same freedom for others, provided that the original source and author(s) are credited.Fábio F. Roxo, Luz E. Ochoa, Gabriel S. C. Silva, Claudio Oliveira

Supplementary material 3 Table S1 Data type: Microsoft Word document Explanation note: Species included in the present study. This dataset is made available under the Open Database License (http://opendatacommons.org/licenses/odbl/1.0/). The Open Database License (ODbL) is a license agreement intended to allow users to freely share, modify, and use this Dataset while maintaining this same freedom for others, provided that the original source and author(s) are credited.Fábio F. Roxo, Luz E. Ochoa, Gabriel S. C. Silva, Claudio Oliveira

Supplementary material 4 Table S2 Data type: Microsoft Word document Explanation note: Primers used in the present study to amplify partial sequences of F-reticulon 4, 16S rRNA, cytochrome oxidase subunit I (COI) and cytochrome B (CytB). This dataset is made available under the Open Database License (http://opendatacommons.org/licenses/odbl/1.0/). The Open Database License (ODbL) is a license agreement intended to allow users to freely share, modify, and use this Dataset while maintaining this same freedom for others, provided that the original source and author(s) are credited.Fábio F. Roxo, Luz E. Ochoa, Gabriel S. C. Silva, Claudio Oliveira

Supplementary material 5 Table S3 Data type: Microsoft Word document Explanation note: DEC models tested to estimate distribution ranges inherited by the descending lineages at each node of the tree. The differences between the models are in the rate of dispersal among adjacent and no adjacent areas. * Represent the model used in the analysis. This dataset is made available under the Open Database License (http://opendatacommons.org/licenses/odbl/1.0/). The Open Database License (ODbL) is a license agreement intended to allow users to freely share, modify, and use this Dataset while maintaining this same freedom for others, provided that the original source and author(s) are credited.Fábio F. Roxo, Luz E. Ochoa, Gabriel S. C. Silva, Claudio Oliveira
