# Supplementary material for: Rhinolekos capetinga: a new cascudinho species (Loricariidae, Otothyrinae) from the rio Tocantins basin and comments on its ancestral dispersal route
Source: Zookeys. 2015 Feb 4;(481):109–30. doi: 10.3897/zookeys.481.8755 (PMC4319103; doi:10.3897/zookeys.481.8755)
Supplement: Supplementary material 1 — Fig. S1 [file zookeys-481-109-s001.pdf]

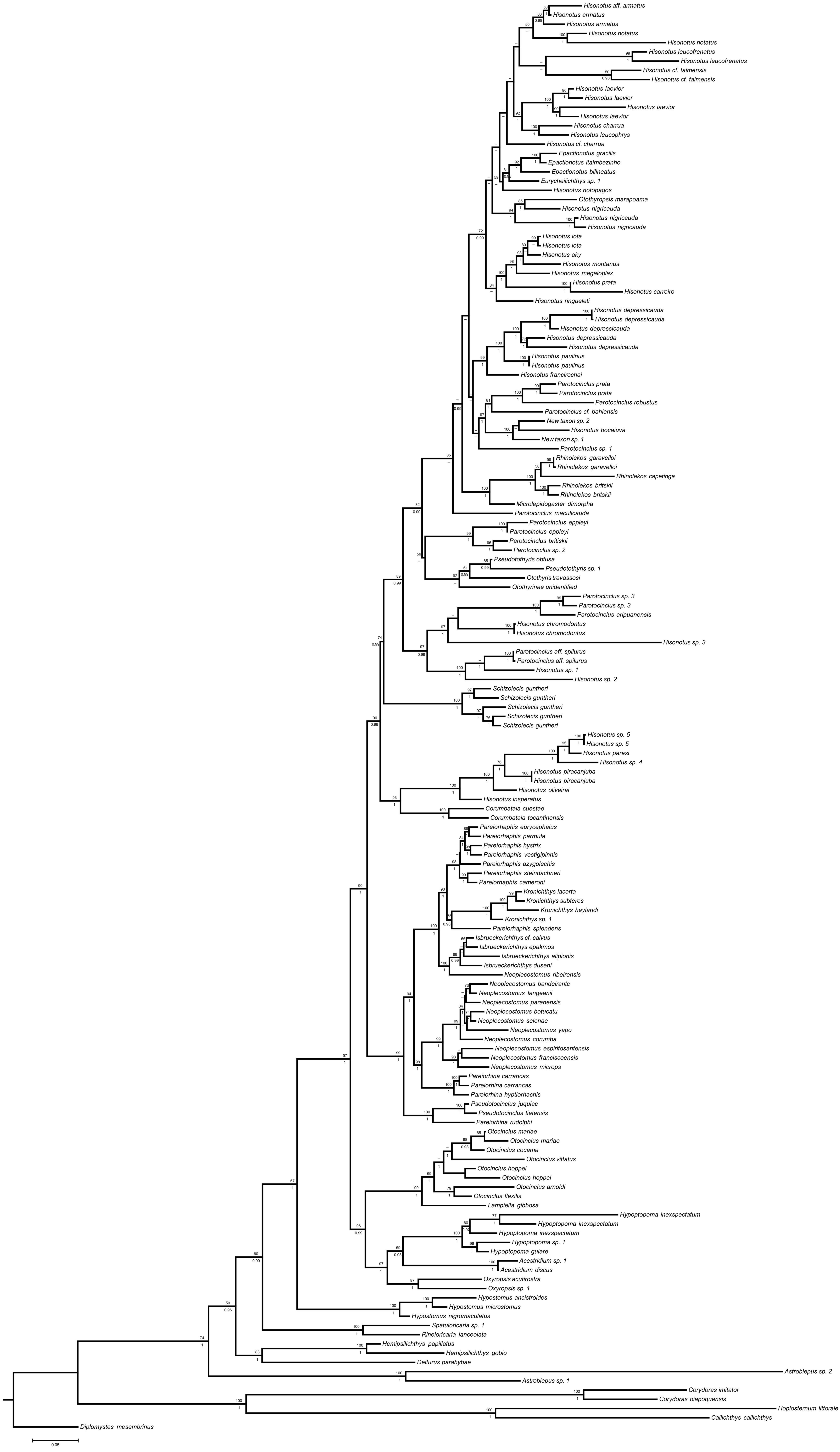

Fig. S1 - Maximum Likelihood tree showing outgroups and interrelationship among species of the subfamily Hypoptopomatinae, Neoplecostominae and Otothyrinae. Numbers above branches are bootstrap values from 1000 bootstrap pseudoreplicates obtained from ML analysis. Bootstrap values below 50% (−) are not shown. Numbers below branches are posterior probabilities obtained in the BI analysis. Posterior probabilities values below 0.95 (−) or when the nodes were not obtained by B analyses are not shown.
