# Supplementary material for: Rhinolekos capetinga: a new cascudinho species (Loricariidae, Otothyrinae) from the rio Tocantins basin and comments on its ancestral dispersal route
Source: Zookeys. 2015 Feb 4;(481):109–30. doi: 10.3897/zookeys.481.8755 (PMC4319103; doi:10.3897/zookeys.481.8755)
Supplement: Supplementary material 2 — Fig. S2 [file zookeys-481-109-s002.pdf]

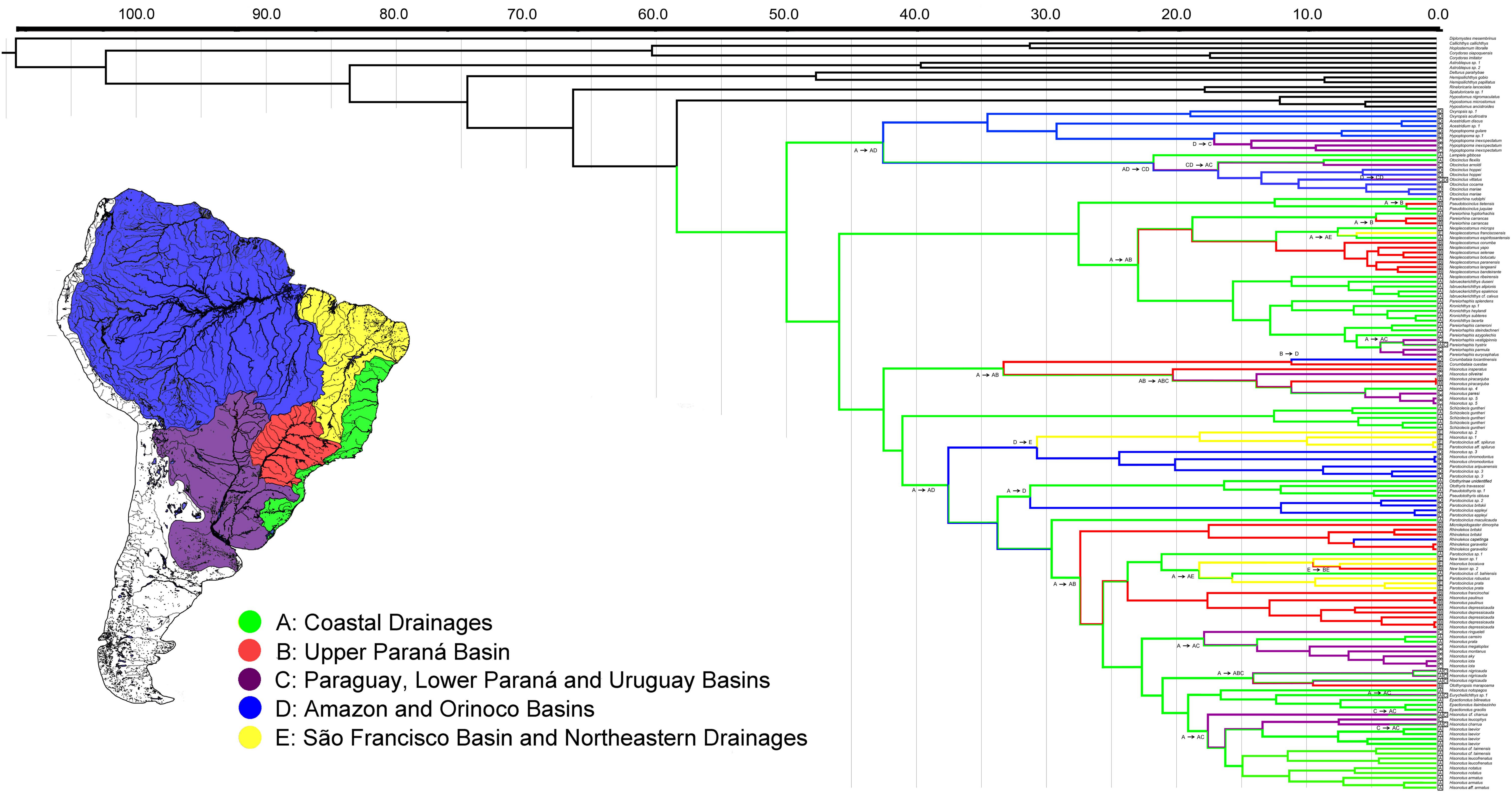

Fig. S2 - Time-calibrated phylogeny for Hypoptopomatinae, Neoplecostominae and Otothyrinae. Tree topology from BEAST analysis of 155 specimens representing 113 loricariid species. Divergence ages calibrated by origins of Siluriformes (120 Ma) and Callichthyidae (55 Ma). Regions: A, Atlantic Coastal Drainages (Green); B, Upper Paraná Basin (Red); C, Paraguay, Lower Paraná and Uruguay Basins (Purple); D, Amazon and Orinoco Basins (Blue); E, São Francisco Basin and Northeastern Drainages (Yellow).
