# Supplementary material for: Rhinolekos capetinga: a new cascudinho species (Loricariidae, Otothyrinae) from the rio Tocantins basin and comments on its ancestral dispersal route
Source: Zookeys. 2015 Feb 4;(481):109–30. doi: 10.3897/zookeys.481.8755 (PMC4319103; doi:10.3897/zookeys.481.8755)
Supplement: Supplementary material 4 — Table S2 [file zookeys-481-109-s004.doc]

**Supplementary table 2.** Primers used in the present study to amplify partial sequences of F-reticulon 4, 16S rRNA, cytochrome oxidase subunit I (COI) and cytochrome B (CytB).

| **Region and Fragment Length** | **Name** | **References** | **Primer Sequence** |
| --- | --- | --- | --- |
| **F-reticulon 4** | Freticul4-D | Chiachio et al. (2008) | 5’-AGG CTA ACT CGC TYT SGG CTT TG-3’ |
|  | Freticul4-R |  | 5’-GGC AVA GRG CRA ART CCA TCT C-3’ |
|  | Freticul4 D2 |  | 5’-CTT TGG TTC GGA ATG GAA AC-3’ |
|  | Freticul4 R2 |  | 5’-AAR TCC ATC TCA CGC AGG A-3’ |
|  | Freticul4 iR |  | 5’-AGG CTC TGC AGT TTC TCT AG-3’ |
| **16S rRNA** | 16Sar | Kocher et al. (1989) | 5’-ACG CCT GTT TAT CAA AAA CAT-3’ |
|  | 16Sbr |  | 5’-CCG GTC TGA ACT CAG ATC ACG T-3’ |
| **COI** | FishF1 | Ward et al. (2005) | 5’-TCA ACC AAC CAC AAA GAC ATT GGC AC-3’ |
|  | FishR1 |  | 5’-TAG ACT TCT GGG TGG CCA AAG AAT CA-3’ |
| **CytB** | L14841 | Oliveira et al. (2011) | 5`-CCA TCC AAC ATC TCA GCA TGA TGA AA 3` |
|  | H15915b |  | 5`-AAC CTC CGA TCT TCG GAT TAC AAG AC 3` |

**Table references**

Kocher, T.D., Thomas, W.K., Meyer, A., Edwards, S.V., Päbo, S., Villablanca, F.X. & Wilson, A. (1989) Dynamics of mito-chondrial DNA evolution in animals: Amplification and sequencing with conserved primers. Proceedings of the National Academy of Sciences, 86, 6196–6200.

Oliveira, C., Avelino, G.S., Abe, K.T., Mariguela, T.C., Benine, R.C., Ort, G., Vari, R.P. & Castro, R.M.C. (2011) Phylogenetic relationships within the speciose family Characidae (Teleostei: Ostariophysi: Characiformes) based on multilocus analysis and extensive ingroup sampling. BMC Evolutionay Biology, 11, 275.

Ward, R.D., Zemlak, T.S., Innes, B.H., Last, P.R. & Hebert, P.D.N. (2005) DNA barcoding Australia’s fish species. Philosoph-ical Transactions of the Royal Society B, 360, 1847–1857.
