# Supplementary material for: Rhinolekos capetinga: a new cascudinho species (Loricariidae, Otothyrinae) from the rio Tocantins basin and comments on its ancestral dispersal route
Source: Zookeys. 2015 Feb 4;(481):109–30. doi: 10.3897/zookeys.481.8755 (PMC4319103; doi:10.3897/zookeys.481.8755)
Supplement: Supplementary material 5 — Table S3 [file zookeys-481-109-s005.doc]

**Supplementary table 3.** DEC models tested to estimate distribution ranges inherited by the descending lineages at each node of the tree. The differences between the models are in the rate of dispersal among adjacent and no adjacent areas. * Represent the model used in the analysis.

|  | **Prediction** | **Dispersal rates between adjacent areas** | **Dispersal rates between no adjacent areas** | **Likelihood** |
| --- | --- | --- | --- | --- |
| M1 | Dispersal between no adjacent areas not permitted | 1.0 | − | lnL = - 255.9 |
| M2 | Dispersal between no adjacent areas permitted | 1.0 | 1.0 | lnL = - 254.8 |
| *M3 | Dispersal between no adjacent areas permitted | 0.5 | 0.0001 | lnL = - 252.5 |
| M4 | Dispersal between no adjacent areas permitted | 0.1 | 0.0001 | lnL = - 263.0 |
